# Music therapy to promote psychological and physiological relaxation in palliative care patients: protocol of a randomized controlled trial

**DOI:** 10.1186/1472-684X-13-60

**Published:** 2014-12-17

**Authors:** Marco Warth, Jens Kessler, Julian Koenig, Alexander F Wormit, Thomas K Hillecke, Hubert J Bardenheuer

**Affiliations:** School of Therapeutic Sciences, SRH University Heidelberg, Maria-Probst-Strasse 3, 69123 Heidelberg, Germany; Centre of Pain Therapy and Palliative Care Medicine, Department of Anaesthesiology, University Hospital Heidelberg, Im Neuenheimer Feld 131, 69120 Heidelberg, Germany; Department of Psychology, The Ohio State University, 175 Psychology Building, 1835 Neil Avenue, Columbus, OH 43210 USA

**Keywords:** Music therapy, Palliative care, Randomized controlled trial, Autonomous functioning, Relaxation, End-of-life care, Quality of life

## Abstract

**Background:**

Music therapy is one of the most frequently used complementary therapies in different palliative care settings. Despite its long tradition and high acceptance by other health-care professionals, evidence on the effectiveness of music therapy interventions for terminally ill patients is rare. Recent reviews and health-care reports consistently point out the need of music therapists to provide an evidence-based rationale for their clinical treatments in this field. Therefore, the present study evaluates the psychological and physiological response of palliative care patients to a standardized music therapy relaxation intervention in a randomized controlled trial.

**Methods/design:**

A sample of 84 participants from a palliative care unit in Heidelberg is randomized to either two sessions of music therapy or two sessions of a verbal relaxation exercise, each lasting 30 minutes. The music therapy sessions consist of live played monochord music and a vocal improvisation, the control group uses a prerecorded excerpt from the mindfulness-based stress reduction program containing no musical elements. Outcome measures include self-report data on subjective relaxation, well-being, pain intensity, and quality of life, as well as continuous recording of heart rate variability and blood volume pulse as indicators of autonomous nervous system functioning.

**Discussion:**

To our knowledge, this study is the first clinical trial in Europe and one of very few randomized controlled trials worldwide to systematically examine the effects of music therapy in palliative care.

**Trial registration:**

German Clinical Trials Register – DRKS00006137

## Background

For more than 35 years, music therapists have been working in different settings of end-of-life care. The Canadian music therapist, Susan Munro, was the first to systematically describe the physical, psychological, social, and spiritual impact of her work with terminally ill patients at the world’s first palliative care unit, founded by Balfour Mount in Montréal [1]. Since then, music therapy has become a substantial part of multidisciplinary palliative care in many industrialized countries. Today, music therapy is one of the most frequently used complementary therapies in US hospices, and is widely accepted by other professional groups in the UK [2, 3]. The German classification of procedures (OPS 8-982 and OPS 8-98e) explicitly recommends the use of music therapy in inpatient and outpatient palliative care treatment [4].

Music therapy is defined as “a systematic process of intervention wherein the therapist helps the client to promote health, using music experiences and the relationships that develop through them as dynamic forces of change” [5]. In clinical palliative care practice, music therapy generally aims at the improvement or maintenance of the patient’s quality of life, including the management of pain and stress, the regulation of negative emotions (e.g. anxiety, anger, and depression), as well as the facilitation of communication and spiritual experiences [6]. Music therapy techniques can be categorized as either active (in case the patient actively participates in the production or reproduction of music) or receptive (where the patient listens to live or prerecorded music). In end-of-life care, techniques typically encompass relaxation or imaginative interventions (receptive), the therapeutic use of songs (active or receptive), and various forms of improvisation (active). Verbal conversation can proceed or complement music therapy interventions but is not an inherent prerequisite for the accomplishment of a music therapeutic contact [7]. In most cases, the interventions are tailored according to the patient’s physical state and psychosocial needs.

Despite its long tradition and positive clinical experiences, limited evidence is available on the effects of music therapy in end-of-life care. The 2010 Cochrane review^a^ included only five trials that implemented (quasi-)randomized controlled designs [8]. Mixed findings emerged with regard to QOL as a study outcome. While Hilliard [9] reports a significant improvement of QOL through music therapy compared to standard care alone, Nguyen [10] did not find a significant effect, supposedly due to a very small sample size. Music therapy has been associated with a reduction of pain [11–14] and anxiety [10, 15], in addition enhancing communication [16] and spiritual well-being [17]. Interestingly, one study [18] reported a decrease in salivary cortisol levels of nine participants after a receptive music therapy session, while other physiological (heart rate, respiration rate) or objective outcomes (e.g. days of survival) did not show significant differences or changes over time [9, 15, 19]. However, many of these results must be interpreted with caution as they stem from studies with high risk of bias due to small sample sizes, missing control group conditions or randomization procedures, as well as inadequate statistical analysis and reporting of data. A review of qualitative findings emphasizes the meaningfulness and important role of music therapy not only for patients, but also for family caregivers and staff members [20].

Although the abovementioned studies suggest that there might be a beneficial effect of music therapy on outcomes relevant for end-of-life care, the decision of how (and whether) to apply music therapy still vastly relies on subjective presumptions and clinical expertise. Current reviews consistently attest a lack of high quality studies, ruling out the possibility to provide an evidence-based recommendation in favor or against the use of music therapy within the field of palliative care [8, 21].

Hence, the general aim of the present study is to improve the body of research on this important but underrepresented topic. More precisely, the study goals are to evaluate the effectiveness of a music therapy relaxation intervention regarding palliative care patients’

1. subjective perception of relaxation, well-being, and pain (i.e. immediate effects on psychological outcomes; primary outcome of this study),
2. autonomous relaxation response indexed by HRV and BVP (i.e. immediate effects on physiological outcomes),
3. health-related quality of life (i.e. medium-term effects).

Therefore, we compare the effects of music therapy to the ones of a control group, using a prerecorded mindfulness exercise without musical elements or therapeutic interaction. We hypothesize improvements over time in both groups, but a superiority of the music therapy intervention on all three aforementioned dimensions.

## Methods/design

The following sections present the details of the research methodology designed to address these objectives. The presented study protocol received IRB approval by the Ethics Committee of the Medical Faculty of Heidelberg University on January 3^rd^ 2013 (S-406/2012).

### Participants and setting

The present study is a collaborative research project in cooperation of the Centre of Pain Therapy and Palliative Care Medicine at the University Hospital Heidelberg and the School of Therapeutic Sciences at the SRH University Heidelberg. Recruitment, interventions, and data collection are carried out at the palliative care unit of the St. Vincentius Hospital in Heidelberg, Germany. All patients are assessed for eligibility according to predefined inclusion and exclusion criteria listed in Table 1.

**Tables 1:** **Inclusion and exclusion criteria**

Inclusion criteria	Exclusion criteria
Palliative care according to OPS 8-892	Terminal phase
Sufficient understanding of German or English language	Cognitive impairments (e.g. brain metastases, ICD 10: C71, C72)
	Apallic syndrome (ICD 10: G93.80)
	Deafness (ICD 10: H90, H91)
	Restlessness and agitation (ICD 10: R45.1)

### Study design and procedures

**Figure 1:**
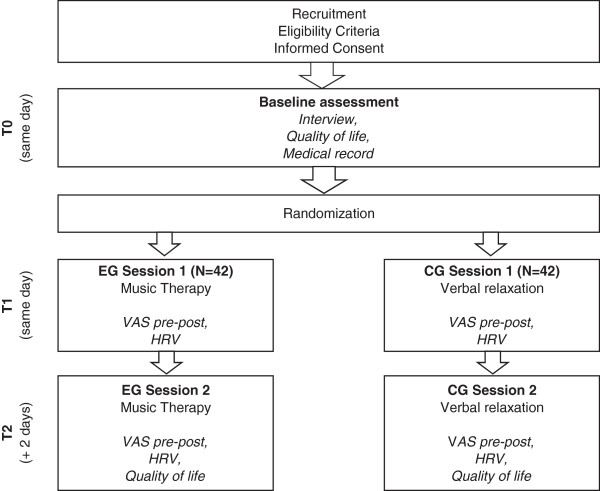
**Study design and procedures.**

The study is designed as a prospective RCT with two parallel arms. Hospitalized patients eligible for study inclusion are contacted at bedside in their rooms and informed about the procedures and objectives of the study. The investigator presents the purpose of the study as to compare the effects and feasibility of two commonly used relaxation interventions for the palliative care context. No information is given about which of the two interventions is the actual experimental condition. If patients agree to participate, the investigator asks them to sign the informed consent sheet, and baseline assessment of the patients’ QOL and previous experience with relaxation techniques is obtained. Following that, the investigator gathers relevant information from the patients’ medical record and opens a serially numbered opaque envelope containing assignment to one of the two study arms (see interventions). We used a computer-based block permutation for the generation of the randomization sequence before the onset of the study. The investigator then returns to the patients’ room with the physiological measuring devices, collects pretest data on relaxation, well-being, and pain using VAS, and starts a 5-minute pretest recording of HRV. Then, either the experimental condition (music therapy) or the control condition (verbal relaxation) is initiated, lasting for twenty minutes. HRV is recorded throughout this procedure. Data collection is complemented by another five minutes of HRV recording and posttest VAS assessment following the intervention. The entire procedure lasts thirty minutes and is repeated two days later, finishing with the follow-up assessment of patients’ QOL. In both groups, the patient rests in a supine position for the entire duration of the session. Figure 1 shows a flow diagram of the study design and procedures.

### Interventions

Patients are randomly assigned to either a music therapy relaxation intervention or a verbal relaxation intervention.

#### Music therapy

The experimental condition is carried out by a professional music therapist with several years of working experience in end-of-life care. The music therapy relaxation technique combines a verbal introduction, soothing sounds played live on a monochord and vocal improvisation. The monochord is designed for the use in therapeutic contexts. Twenty-one equally tuned strings and 3 bass strings are placed on a rectangular wooden corpus. A slow and even play of the strings creates a full and atmospheric monochrome sound that is perceived as calming or relaxing. A recent qualitative study found the body tambura (an instrument with similar characteristics) to elicit pleasant images and visualizations in palliative care patients [22]. A pilot EEG study with oncological patients showed that monochord music may be associated with an increase in posterior theta activity and a decrease in alpha band and mid-frontal beta-2 band activity, most likely reflecting a relaxation response [23]. In another pilot study, patients in oncological rehabilitation reported to be more balanced, less nervous, and less exhausted after listening to monochord music [24].

After the HRV pretest recording, the experimenter leaves the room for the remainder of the intervention. The music therapist briefly explains the process and principle of the intervention. The actual treatment starts with a brief body scan and breathing exercise accompanied by low and gentle sounds on the monochord. The therapist then initiates a vocal improvisation in an ionian or mixolydian mode and raises volume and dynamics of the musical play, adapted to the patient’s breathing. After 10 minutes, the improvisation reaches its peak and the therapist gradually and slowly reduces the musical parameters of the instrumental and vocal play. The patient is asked, gently to return his or her attention to the present situation. A brief feedback conversation gives the participant the opportunity to reflect on his experiences during the music therapy intervention. After another five minutes, the therapist leaves the room and the investigator returns for posttest outcome assessment and debriefing.

#### Verbal relaxation

The verbal relaxation intervention uses a 20-minute excerpt from Jon Kabat-Zinn and Ulrike Grossmann’s CD “Stressbewältigung durch die Praxis der Achtsamkeit”^b^ [25], adopted from the *mindfulness-based stress reduction* (MBSR) program. MBSR is a structured 8–10 weeks group program that has shown to effectively improve the physical and mental health of participants from different medical and non-medical contexts by training participants’ self-awareness and mindfulness [26].

The control condition’s time-frame is identical to the experimental condition (5 min. baseline → 20 min. intervention → 5 min. postline). The standardized application of the intervention utilizes a conventional MP3 player and stereo headphones and therefore, requires no additional personnel. The investigator stays silently inside the room, in case the patient expresses unease or wants to cancel the intervention. The prerecorded MBSR track lasts twenty minutes and contains a body scan and breathing exercise, spoken by a soft, female voice. After completion, the investigator asks the participant to remain in his position for the 5-minute posttest HRV recording and VAS assessment.

**Figure 2:**
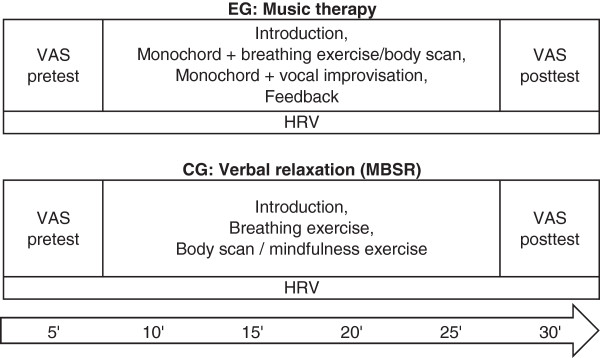
**Intervention procedures and time course.**

Figure 2 provides an overview of the procedures and time course of the experimental and control condition.

### Outcomes

#### VAS (primary outcome)

Patients’ subjective experience of immediate short-time effects of the intervention is measured via three different VAS, assessing relaxation *(-5 = “very tense”* to *+5 = “very relaxed”)*, general well-being *(−5 = very bad* to *+5 = very good)*, and pain intensity *(0 = no pain at all* to *10 = worst pain possible)*. The scales’ endpoints depict good vs. bad smileys and a color code from red to green for better visualization of the valences. Subjective ratings are assessed immediately before and after each session.

#### HRV/BVP

During the 30-minutes intervention we collect continuous data on the intervals between successive heartbeats in milliseconds (i.e. HRV). For reason of feasibility and lower patient burden, we decided to use a BVP finger sensor with a sampling rate of 128 SPS for the least invasive signal processing, instead of a high-resolution ECG signal that would require electrode placement. A NeXus-16® interface sends the BVP signal to the BioTrace + ® software on a laptop computer via Bluetooth. BioTrace® records raw data of all sessions and exports IBI text files for each participant and session. Kubios v2.1® transforms these tables into HRV parameters based on six successive 5-minutes-samples for further statistical analyses with IBM SPSS Statistics 20®.

Despite the derivation of HRV parameters, BVP signals offer the possibility to measure vasodilation/vasoconstriction as a second set of markers on autonomous functioning. A shift to parasympathetic dominance is expected to manifest in an increase in the BVP amplitude. In accordance to the HRV analysis, we will calculate mean values and standard deviations for 5-minutes-intervals for each patient and subject.

#### EORTC QLQ-C15-PAL

The EORTC QLQ-C30 is one of the most frequently used questionnaires to measure health-related quality of life in cancer patients. Its short version (EORTC QLQ-C15-PAL) was specifically designed for the application in palliative care settings [27]. The questionnaire consists of 15 items measuring nine of the original instrument’s subscales *(global health status/quality of life, physical functioning, emotional functioning, fatigue, nausea/vomiting, pain, dyspnoea, insomnia, appetite loss, constipation)* on fourteen 4-point scales *(0 = “not at all”, 1 = “a little”, 2 = “quite a bit”, 3 = “very much”),* in addition to the patient’s global health status/quality of life on a 7-point scale *(1 = very poor, 7 = excellent).* The EORCT QLQ-C15-PAL is administrated once at baseline and a second time after completion of both sessions. We use the German translation provided at the EORTC’s website [28].

### Data analysis

#### Sample size calculation

Statistical power calculations for this study are adjusted for immediate short-time effects on self-report VAS data (i.e. the primary outcome). Bradt and Dileo’s [8] findings suggest that medium sized effects are most likely to be expected for the impact of music therapy on psychosocial outcomes in palliative care. Recent articles [29, 30] on sample size calculations emphasize the superiority of ANCOVA models for analyzing the results of randomized, controlled trials over the repeated-measures ANOVA approach. The inclusion of pretest values as a covariate in these models increases statistical power (i.e., the likelihood to identify statistically significant effects).

Assuming statistical power of (1-β) = .80, type-I error probability of α = .05, and a correlation between covariate and outcome of ρ = .6, a total sample size of *N* = 84 is required to detect medium sized effects between groups [29]. With an expected drop-out rate of 10%, approximately 92 participants need to be recruited for this study. The risk of attrition bias will be addressed in the course of intention-to-treat analyses.

#### Statistical analyses

We will test for significant differences between groups at baseline using independent samples t-tests, with α = .20 to reduce type-II error probability. For all study hypotheses tests, type-I error probability is set to α = .05. VAS and QOL data will be analyzed with ANCOVA models, where posttest values serve as criteria, pretest scores as covariates and group assignment as a fixed predictor, respectively. We will run repeated-measures ANOVA on physiological data (HRV/BVP) to test for polynomial trends over time, main effects of group allocation, and interaction effects.

## Discussion

This paper presents the study protocol of a randomized controlled trial on the psychological and physiological effects of a music therapy relaxation intervention for terminally ill patients in palliative care.

One crucial aspect of clinical trials in palliative care research is the design of an adequate control group condition. Different authors took controversial stands regarding the ethical justifiability of applying rigorous, quantitative research methodology in general, and random assignment to study arms in particular to populations of terminally ill patients [31–33]. Withholding potentially effective treatments due to randomization can be particularly problematic. Therefore, we decided to include an active control treatment instead of a no treatment or treatment as usual condition. The experimental and control group intervention share one possible factor of therapeutic change, namely the concept of mindfulness, which is present in both interventions. Hence, if significant between-group differences are to appear, they need to be attributed to factors unique to the music therapy treatment. These are associated with social aspects or the therapeutic relationship on the one hand and characteristics of the live-played music itself, both inherent parts of the definition of music therapy as a health-care profession [5]. Hence, we believe this study design to be appropriate to evaluate the effectiveness of the music therapy relaxation intervention described above. In the course of the analysis and discussion of the study results, we will have to address the question of how the comparison of two active treatments affects the interpretation of our findings. We discuss methodological challenges associated with the implementation of clinical controlled trials on the effectiveness of music therapy in palliative care in more detail in a recently published article [34].

As the study is not supported by external sponsors, no resources for additional study personnel are available. Hence, we expect a long data collection period. As with all interventional studies in palliative care, high drop-out rates are likely to become a particular issue that must be handled in the course of sensitivity analysis.

The research methodology of this study contains some trade-offs and subjective decisions that were discussed intensively in our team (e.g. the decision for a less precise but also less invasive method of HRV measurement). We are confident to have set up a both ethically justifiable and feasible research design that provides high methodological rigor capable of producing valid and reproducible results. To our present knowledge, this study will be the first European RCT on the effects of music therapy in palliative care.

## Endnotes

^a^Withdrawn from the Cochrane database of systematic reviews in March, 2014.

^b^English translation: “*Reducing stress by practicing mindfulness”.*

## Authors information

MW, MA, is a research associate at the School of Therapeutic Sciences, SRH University Heidelberg. He is a doctoral candidate at the Medical Faculty of Heidelberg University and a research assistant at the Centre of Pain Therapy and Palliative Care Medicine, Department of Anaesthesiology, University Hospital Heidelberg.

JKe, MD, is a physician at the Centre of Pain Therapy and Palliative Care Medicine, Department of Anaesthesiology, University Hospital Heidelberg. He specialized in anaesthesiology, pain therapy, emergency medicine, and palliative care medicine.

JKo, Dr. sc. hum., is a post-doctoral researcher at the Department of Psychology, The Ohio State University.

AFW, Dr. sc. hum., is Professor of Clinical Music Therapy and vice dean of the School of Therapeutic Sciences, SRH University Heidelberg.

TKH, Dr. sc. hum., is Professor of Clinical Psychology and dean of the School of Therapeutic Sciences, SRH University Heidelberg, Germany.

HJB, MD, is a physician specialized in anaesthesiology, pain therapy, and palliative care medicine. He is University Professor at Heidelberg University and Head of the Centre of Pain Therapy and Palliative Care Medicine, Department of Anaesthesiology, University Hospital Heidelberg. He is Chief of the University Palliative Care Medicine at St. Vincent Hospital, Heidelberg.

## Abbreviations

AN(C)OVA: Analysis of (co-)variance
BVP: Blood volume pulse
CG: Control group
EG: Experimental group
EORTC: European Organization for Research and Treatment of Cancer
HRV: Heart rate variability
IBI: Inter-beat-interval
ICD: International Statistical Classification of Diseases and Related Health Problems
MBSR: Minfulness-based stress reduction
OPS: Operationen- und Prozedurenschlüssel (German procedure classification)
QOL: Quality of life
RCT: Randomized controlled trial
VAS: Visual analogue scale.
